# Mining the FAERS database reveals new safety signals for 120 mg denosumab in oncology practice

**DOI:** 10.1371/journal.pone.0342188

**Published:** 2026-02-02

**Authors:** Shaohuan Lu, Dongxiao Wang, Caiming Xiong, Jialing Liang, Yang Li, Canhua Liang, Qianxi Chen, Guangyi Meng

**Affiliations:** 1 Department of Pharmacy, The First People’s Hospital of Yulin, Yulin, Guangxi, China; 2 Department of Pharmacy, Yulin Maternal and Child Health Hospital, Yulin, Guangxi, China; 3 School of Pharmacy, Guangxi Medical University, Nanning, Guangxi, China; 4 Yulin Campus, Guangxi Medical University, Yulin, Guangxi, China; Showa University, JAPAN

## Abstract

**Objective:**

To detect and quantify adverse drug event (ADE) signals linked to 120 mg denosumab in the real world.

**Methods:**

We queried the US Food and Drug Administration Adverse Event Reporting System (FAERS) for reports on 120 mg denosumab, standardized, and categorized these events with Preferred Terms (PT) and System Organ Classes (SOC) from MedDRA version 27.1. Disproportionality analysis was performed using the reporting odds ratio (ROR) and Bayesian confidence propagation neural network (BCPNN).

**Results:**

We identified 10,963 ADE reports for 120 mg of denosumab. Among 10 963 reports, 41.6% of patients were ≥ 65 years old, 37.1% were from the United States, and 17.7% were from Japan; 77.0% were serious. Data mining revealed 294 positive disproportionality signals across 27 SOCs. The highest signal proportions were for neoplasms (15.99%), musculoskeletal disorders (13.27%), infections (11.56%), gastrointestinal disorders (10.88%), and procedural complications (10.20%). The most frequent ADEs were osteonecrosis of the jaw (n = 3,082, 28.11%), death (n = 803, 7.32%), hypocalcemia (n = 799, 7.29%), and fatigue (n = 282, 2.57%). The strongest signals by ROR were bone giant cell tumor (7888.08), bone giant cell tumor malignant (6502.96), osteonecrosis of the jaw (396.57), and bone giant cell tumor benign (312.37). Thirty-two unlisted events were identified, including exostosis of jaw, osteosarcoma, tooth fracture, and bone lesions.

**Conclusions:**

Clinicians should maintain vigilance for both established toxicities and these 32 emerging signals to ensure the safety of patients receiving 120 mg of denosumab for oncological purposes.

## 1. Introduction

Denosumab is a human IgG2 monoclonal antibody that binds the receptor activator of nuclear factor-κB ligand (RANKL) with high specificity and affinity, thereby preventing RANKL from engaging its cognate receptor (RANK) [1–3]. This blockade suppresses osteoclast maturation, function, and survival. The drug is approved for osteoporosis [4,5], giant cell tumors of the bone [6], and skeletal-related events (SREs) arising from solid tumor bone metastases [7]. In the treatment of osteoporosis, it can effectively reduce bone resorption, increase bone density, and significantly reduce the risk of fracture. The clinical efficacy is significant, especially in postmenopausal women with osteoporosis [8].

The indications for denosumab at 60 mg and 120 mg doses differ. Denosumab 60 mg is indicated for osteoporosis in postmenopausal women at high risk of fracture, osteoporosis in men at high risk of fracture, and glucocorticoid-induced osteoporosis in patients at high risk of fracture. Denosumab 120 mg is used to treat bone metastases from solid tumors, multiple myeloma, and giant cell tumors of the bone. In the treatment of bone metastases from solid tumors, it can delay the occurrence of bone-related events and improve the quality of life. Its clinical application is becoming increasingly widespread, and its safety has received increasing attention. Current studies on the adverse drug events (ADEs) of denosumab include early randomized controlled trials [3,9–11], literature reports [12–14], clinical observational studies [15–20], and signal detection (the systematic identification of potential safety concerns through disproportionality analysis of adverse event reports) of denosumab ADRs based on real-world data [21–23]. Previous signal detection studies based on the US Food and Drug Administration Adverse Event Reporting System (FAERS) database have either combined the analysis of 60 mg and 120 mg of denosumab [21,22] or focused solely on the 60 mg dose for osteoporosis indications [23].Currently, there is a lack of real-world ADE signal assessment for the 120 mg tumor-specific dose, making it difficult to provide precise evidence-based support for its clinical use in tumor therapy. Unlike previous FAERS investigations that either pooled all denosumab doses or focused solely on the 60 mg formulation, our oncology-specific 15-year analysis provides the most comprehensive safety landscape to date for the 120 mg dosage.

We used both the Reporting Odds Ratio (ROR) and Bayesian Confidence Propagation Neural Network (BCPNN) methods to detect signals, ensuring robustness and sensitivity. By dissecting drug-related adverse event signals, we aim to guide the prevention and management of adverse events during the safe clinical use of 120-mg denosumab in antitumor therapy and to inform treatment selection and evidence-based prescribing decisions.

## 2. Materials and methods

### 2.1. Data source

This study utilized the FAERS database, a global pharmacovigilance resource with voluntary reports from several countries. For data mining and statistical analysis, raw ASCII data packages were downloaded for this study from the US FDA website:https://fis.fda.gov/extensions/FPD-QDE-FAERS/FPD-QDE-FAERS.html. ADE reports and risk signal data from the first quarter of 2009 to the third quarter of 2024 were extracted based on the market release date of denosumab in the 120 mg dosage.

### 2.2. Data processing procedure

We sorted through 2,196,449 patient records in the FAERS database and utilized the DEMO table fields PRIMARYID (a unique report identifier), CASEID (a case number that can group multiple follow-up reports), and FDA_DT (the date the FDA received the report). Following the FDA deduplication rule, we retained the report with the most recent FDA_DT when several records shared the same CASEID. If both CASEID and FDA_DT were identical, we retained the record with the largest PRIMARYID. An example is provided in S1Table.

**Fig 1:**
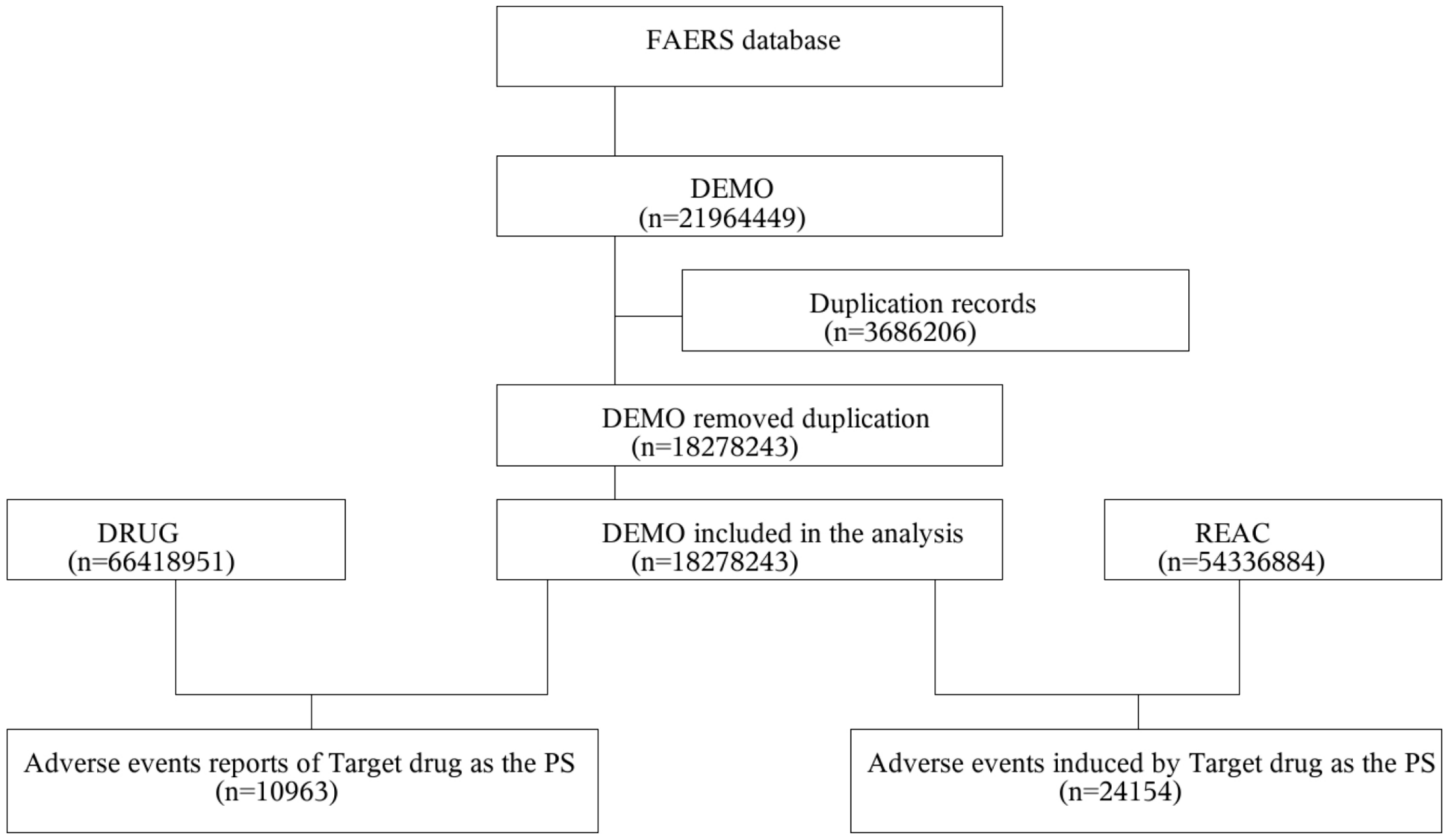
Flowchart depicting the selection of the study population.

As Xgeva was the only denosumab product licensed for cancer-related indications during the study period and Wyost and Xbyrk were approved after the dataset end date, these products were neither retrieved by our search nor included in the ADE analysis. We queried the database using the generic name “Denosumab (Xgeva)” and the trade name “Xgeva” as target drugs, bone-related cancers as indication, and include only ADRs reports where Denosumab (Xgeva) is the primary suspected drug (PS) to improve accuracy [24]. After data cleaning, 10,963 denosumab-related ADE reports (involving 24,154 adverse events) were included in the subsequent analysis (Fig 1). The extracted data were standardized using the preferred terms (PTs) from the Medical Dictionary for Regulatory Activities (MedDRA 27.1) and then mapped to their corresponding system organ class (SOC) terms. MedDRA is a clinically validated, internationally harmonized terminology developed by the International Council for Harmonization of Technical Requirements for Pharmaceuticals for Human Use (ICH). By standardizing medical concepts across regulators, industry, and academia, the MedDRA ensures consistent signal detection, safety profiling, and communication of medicine-related risks worldwide.

### 2.3. Signal detection

The Reporting Odds Ratio (ROR) and Bayesian Confidence Propagation Neural Network (BCPNN) methods were employed to analyze the collected denosumab ADE reports. ROR is a classic frequency-based measure of association. It calculates the reported odds ratio of the association between a specific drug and a specific adverse event using a simple 2 × 2 contingency table. The ROR method has the advantage of eliminating bias and offering high sensitivity, but it is prone to false positives [25]. BCPNN is a complex algorithm based on the Bayesian statistical theory. This makes the results more stable and specific [26,27]. Based on the relevant literature [25], this study incorporated both ROR and BCPNN methods to analyze and evaluate post-marketing ADE signals for denosumab 120 mg. The ROR was calculated using a 2 × 2 contingency table (Table 1 [28,29]). Based on the thresholds in Table 2 [28,30–32], each PT was assessed, and an ADE signal was generated only if it met the two criteria (“a ≥ 3, lower limit of 95% CI ≥ 1for ROR” and “IC025 > 0 for BCPNN”). The emergence of an ADE signal indicates a statistical association between the ADE and drug, with a larger signal value representing a stronger correlation [29,33]. The specific grading criteria for the signal strength are presented in Table 3 [33]. We excluded signals that were clearly irrelevant to the drugs, such as off-label uses and product use in unapproved indications [34,35].

**Table 1:** Two-by-two contingency table for disproportionality analysis.

	Target adverse drug event	Other adverse drug events	Sums
Denosumab	a	b	a + b
Other drugs	c	d	c + d
Sums	a + c	b + d	n = a + b + c + d

**Table 2:** Tow major algorithms used to assess potential associations between Denosumab and ADEs.

Algorithms	Equation	Criteria
ROR	ROR = ad/ bc	a ≥ 3, Lower limit of 95% CI ≥ 1
	95% CI = e ln (ROR) ± 1.96(1/a + 1/b + 1/c + 1/d) ^ 0.5	
BCPNN	IC = log 2a (a + b + c + d)/ [(a + c) (a + b)]	IC025 > 0
	95% CI = E(IC) ± 2[V(IC)] ^ 0.5	

Notes: IC: Information Component; IC025:lower limit of 95% CI of the IC.

**Table 3:** Graduated Signal-Strength Criteria for the Reporting Odds Ratio (ROR) and Bayesian Confidence Propagation Neural Network (BCPNN) Methods.

Item	Weak signal	Moderate-strength signal	High-strength signal
ROR	1 < ROR-1.96SE < 50	50 ≤ ROR-1.96SE < 1000	ROR-1.96SE ≥ 1000
BCPNN	0 < IC-2SD ≤ 1.5	1.5 < IC-2SD ≤ 3.0	IC-2SD > 3.0

### 2.4. Define serious ADE report

The assessment of serious ADE reports was based on the “OUTC_COD” column in the “outc file”, evaluated according to FDA standard criteria: death, life-threatening condition, hospitalization, disability, congenital anomaly, or required intervention.

### 2.5. Ethics statement

This study used signal de-identified data from the FDA Adverse Event Reporting System, a publicly accessible database. All analyses were conducted in strict compliance with the FDA’s publicly available data-use terms and the FAERS Data Release Agreement. No identifiable patient information was accessed or downloaded at any stage of the research. Consequently, institutional review board (IRB) approval was not required.

## 3. Results

### 3.1. Basic characteristics of denosumab-related ADE

A total of 10,963 ADE reports with 120 mg of denosumab as the primary suspected drug were included in the US FAERS database from the first quarter of 2009 to the third quarter of 2024, covering 83 quarters of data collection. More reports involved female patients (47.92%) than male patients (40.31%); patients aged ≥ 65 years represented 41.64% of the reports; and the majority of reports were from the United States (37.12%) and Japan (17.69%). Serious ADE reports accounted for 77.02% of the total reports. ADEs can occur at different time intervals post-administration. See Table 4. Among 10693 cases reporting at least one ADE, 3130 (29.27%) had sufficient data to calculate the time-to-onset; of the 8444 serious ADE reports, 2895 (34.28%) could be dated. Both overall and serious ADEs exhibited an ‘early-onset plus ultra-delayed’ pattern: nearly 40% of events (37.25% for total VS 40.97% for serious) were reported more than one year after drug initiation. See S2 Table.

**Table 4:** Basic characteristics of adverse drug event reports associated with denosumab.

Item	Number of case (%)
Sex	
Male (%)	4653(42.44)
Female (%)	5254(47.92)
Not specified (%)	1056(9.63)
Age	
< 18(%)	75(0.68)
18-64(%)	3031(27.65)
≥ 65(%)	4565(41.64)
Not specified (%)	3292(30.03)
Country of ADE Onset	
United States (%)	3435(31.33)
Japan (%)	1917(17.49)
Not specified (%)	838(7.64)
Germany (%)	706(6.44)
France (%)	534(4.87)
Canada (%)	501(4.57)
Netherlands (%)	381(3.48)
Other countries (%)	2651(24.18)
Serious Reports	
Serious (%)	8444(77.02)
Non-serious (%)	2519(22.98)
Outcome	
Other serious medically important event (%)	6559(59.83)
Hospitalization (%)	2677(24.42)
Death (%)	1325(12.09)
Disability (%)	390(3.56)
Life-threatening condition (%)	200(1.82)
Required intervention (%)	12(0.11)
Congenital anomaly (%)	3(0.03)
Time-to-onset (TTO) (days)	
0-90d (%)	1147(10.46)
91-180d (%)	316(2.88)
181-360d (%)	481(4.39)
> 360d (%)	1186(10.82)
Missing or invalid values (< 0) (%)	7833(71.45)


Notes: Time-to-onset (TTO) was outlined as the period of time between the ADE occurrence date (EVENT DT in the DEMO file) and the beginning date of drug use (START DT in the THER file). “Missing or invalid values” denote reports in which the onset time could not be calculated because the EVENT_DT or the START_DT was absent, non-numeric, or logically inconsistent (e.g., EVENT_DT earlier than START_DT); they are unrelated to missing demographic variables such as age or sex.

### 3.2. Denosumab ADR signals involving SOCs

**Fig 2:**
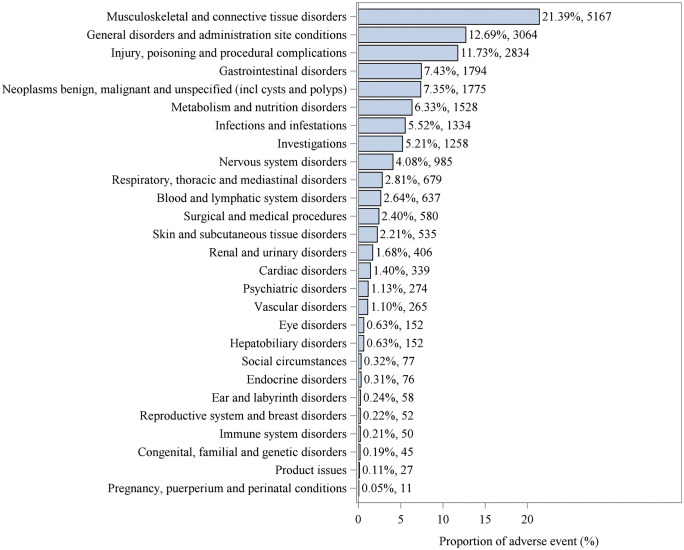
Proportion of adverse events by SOCs.

Among the 10,963 ADE reports, 294 positive ADE signals were identified, which encompassed 27 different SOCs. The SOCs were ranked by the number of occurrences, with the most affected being musculoskeletal and connective tissue disorders (21.39%), general disorders and administration site conditions (12.69%), injury, poisoning, and procedural complications (11.73%), gastrointestinal disorders (7.43%), and neoplasms benign, malignant, and unspecified (including cysts and polyps) (7.35%). See Fig 2 and Table 5.

**Table 5:** Distribution of Positive Safety Signals for Denosumab-Associated Adverse Drug Events Across SOCs.

No.	SOC	Positive Signals	Proportion (%)
1	Musculoskeletal and connective tissue disorders	39	13.27
2	General disorders and administration site conditions	12	4.08
3	Injury, poisoning and procedural complications	30	10.20
4	Gastrointestinal disorders	32	10.88
5	Neoplasms benign, malignant and unspecified (incl cysts and polyps)	47	15.99
6	Metabolism and nutrition disorders	14	4.76
7	Infections and infestations	34	11.56
8	Investigations	26	8.84
9	Nervous system disorders	9	3.06
10	Respiratory, thoracic and mediastinal disorders	6	2.04
11	Blood and lymphatic system disorders	10	3.40
12	Surgical and medical procedures	16	5.44
13	Skin and subcutaneous tissue disorders	1	0.34
14	Renal and urinary disorders	3	1.02
15	Cardiac disorders	2	0.68
16	Psychiatric disorders	0	0.00
17	Vascular disorders	1	0.34
18	Hepatobiliary disorders	1	0.34
19	Eye disorders	0	0.00
20	Social circumstances	4	1.36
21	Endocrine disorders	4	1.36
22	Ear and labyrinth disorders	0	0.00
23	Reproductive system and breast disorders	0	0.00
24	Immune system disorders	0	0.00
25	Congenital, familial and genetic disorders	2	0.68
26	Product issues	1	0.34
27	Pregnancy, puerperium and perinatal conditions	0	0.00
28	Sums	294	100

Notes:

(1) The number of positive signals refers to the number of signals detected using the preferred term (PT) name under the specified organ system classification (SOC). Note that the number here refers to the number of PT types, not the number of PT.

(2) Signal proportion under organ system classification (SOC) = number of signals under organ system classification (SOC)/ total number of signals for the target drug.

(3) Occurrence refers to the number of adverse events involving organ system classification (SOC).

(4) Proportion = number of adverse events involving the organ system classification (SOC)/ total number of adverse events.

### 3.3. Top 30 ADE signals for Denosumab

Of the 320 ROR-positive signals, 294 were concordantly flagged by BCPNN, yielding an overlap of 91.9% (S3 and S4 Tables). The 294 positive signals were ranked by frequency of occurrence, and the top 30 PTs were identified. The highest frequency was reported for osteonecrosis of the jaw (3082 cases, 28.11%), followed by death (803 cases, 7.32%), hypocalcemia (799 cases, 7.29%), fatigue (282 cases, 2.57%), bone pain (231 cases, 2.11%), and nausea (230 cases, 2.10%). See Table 6.

**Table 6:** Top 30 Ranked Risk Signals for Denosumab by Report Frequency.

No.	PT	Cases	ROR (95% CI)	IC(IC-2SD)
1	Osteonecrosis of jaw	3082	396.57(380.92-412.86)	8.23(8.04)
2	Death	803	2.45(2.29-2.63)	1.27(1.16)
3	Hypocalcemia	799	119.48(111.15-128.43)	6.78(6.49)
4	Fatigue	282	0.93(0.83-1.05)	−0.10(−0.27)
5	Bone pain	231	10.06(8.83-11.45)	3.31(3.07)
6	Nausea	230	0.74(0.65-0.85)	−0.42(−0.61)
7	Pain	224	0.91(0.80-1.04)	−0.13(−0.32)
8	Bone giant cell tumor benign	219	7888.08(5958.71-10442.2)	10.77(7.35)
9	Metastases to bone	191	31.84(27.59-36.75)	4.96(4.54)
10	Tooth extraction	184	37.33(32.26-43.21)	5.19(4.72)
11	Diarrhea	177	0.71(0.62-0.83)	−0.48(−0.70)
12	Tooth disorder	177	19.81(17.08-22.98)	4.29(3.93)
13	Arthralgia	169	1.06(0.91-1.24)	0.09(−0.13)
14	Pain in jaw	167	14.77(12.68-17.20)	3.87(3.53)
15	Dyspnea	167	0.75(0.64-0.87)	−0.41(−0.63)
16	Neutropenia	162	3.14(2.69-3.67)	1.64(1.40)
17	Disease progression	160	3.53(3.02-4.13)	1.81(1.56)
18	Asthenia	156	1.05(0.90-1.23)	0.08(−0.16)
19	Osteomyelitis	151	20.79(17.71-24.42)	4.36(3.95)
20	Pyrexia	147	1.08(0.92-1.27)	0.11(−0.13)
21	Anemia	142	1.87(1.59-2.21)	0.90(0.65)
22	Vomiting	134	0.74(0.62-0.87)	−0.44(−0.68)
23	Back pain	127	1.38(1.16-1.64)	0.46(0.20)
24	Pain in extremity	127	1.07(0.90-1.28)	0.10(−0.15)
25	Malaise	124	0.70(0.59-0.84)	−0.50(−0.76)
26	Atypical femur fracture	122	115.17(95.97-138.21)	6.77(5.59)
27	Breast cancer metastatic	121	29.24(24.43-35.00)	4.85(4.29)
28	Fall	119	0.91(0.76-1.09)	−0.13(−0.40)
29	Pneumonia	116	0.87(0.73-1.05)	−0.19(−0.46)
30	Decreased appetite	114	1.20(1.00-1.45)	0.27(−0.01)

Note: This table is ranked solely by adverse event frequency (number of cases); non-positive signals are displayed.

The 294 positive signals were also ranked by signal strength (using the ROR method), with the strongest signal attributed to bone giant cell tumor, followed by bone giant cell tumor malignant, osteonecrosis of jaw, bone giant cell tumor benign, radiotherapy to bone, metastases to the pituitary gland, and jaw fistula. See Table 7.

**Table 7:** Top 30 ranked risk signals for Denosumab by positive signal strength.

No.	PT	Cases	ROR (95% CI)	IC(IC-2SD)
1	Bone giant cell tumor	219	7888.08(5958.71-10442.2)	10.77(7.35)
2	Bone giant cell tumor malignant	26	6502.96(3046.92-13879.1)	10.71(4.00)
3	Osteonecrosis of jaw	3082	396.57(380.92-412.86)	8.23(8.04)
4	Bone giant cell tumor benign	5	312.37(122.57-796.08)	8.10(1.30)
5	Radiotherapy to bone	3	293.33(88.07-977.03)	8.02(0.44)
6	Metastases to pituitary gland	4	264.59(93.88-745.67)	7.89(0.93)
7	Jaw fistula	10	212.22(110.95-405.91)	7.60(2.48)
8	Periorbital abscess	3	164.55(50.95-531.43)	7.26(0.47)
9	Peri-implantitis	5	158.38(63.95-392.29)	7.21(1.31)
10	Atypical fracture	30	144.63(99.96-209.25)	7.09(4.13)
11	Osteonecrosis of external auditory canal	8	135.30(66.28-276.19)	7.00(2.09)
12	Hypocalcemia	799	119.48(111.15-128.43)	6.78(6.49)
13	Blood 1,25-dihydroxycholecalciferol increased	3	116.32(36.44-371.27)	6.79(0.47)
14	Hypocalcemic seizure	3	116.32(36.44-371.27)	6.79(0.47)
15	Atypical femur fracture	122	115.17(95.97-138.21)	6.77(5.59)
16	Osteosarcoma	17	114.53(70.34-186.48)	6.77(3.26)
17	Hungry bone syndrome	6	99.23(43.80-224.78)	6.57(1.60)
18	Sequestrectomy	27	93.65(63.71-137.65)	6.49(3.87)
19	Malignant transformation	15	92.72(55.31-155.42)	6.48(3.04)
20	Exposed bone in jaw	81	89.62(71.75-111.95)	6.43(5.07)
21	Hypercalcemia of malignancy	8	84.48(41.70-171.15)	6.35(2.05)
22	Metastases to muscle	5	80.90(33.15-197.45)	6.29(1.29)
23	Abscess jaw	33	78.05(55.15-110.46)	6.24(4.06)
24	Tooth resorption	6	74.56(33.05-168.17)	6.17(1.58)
25	Cancer of endometrium metastatic	4	64.26(23.78-173.62)	5.97(0.92)
26	Jaw operation	23	63.60(42.01-96.27)	5.95(3.53)
27	Oral cavity fistula	13	63.58(36.63-110.35)	5.95(2.75)
28	Dental fistula	10	57.53(30.71-107.79)	5.81(2.34)
29	Dental prosthesis user	3	55.30(17.59-173.87)	5.75(0.46)
30	Periodontitis	46	53.72(40.09-71.99)	5.71(4.22)

### 3.4. New suspected ADEs for Denosumab

The 294 denosumab ADE-positive signals were classified according to the ROR and BCPNN strength-grading standards outlined in Table 2. This study recorded only ADEs with moderate strength signals or above, specifically those meeting the criteria of IC₀₂₅ > 1.5, while excluding signals unrelated to the drug itself, such as those associated with social environments, product issues, and various surgical and medical procedures. A total of 89 ADE signals were identified, of which 57 ADEs were explicitly mentioned or associated with the 120 mg dosage in the product labeling, with 32 new suspected ADE signals. See Table 8.

**Table 8:** Suspected adverse drug events associated with Denosumab.

SOC	PT (n)
Neoplasms benign, malignant and unspecified (incl cysts and polyps)	Bone giant cell tumor (219), Metastases to bone (191), Breast cancer metastatic (121), Metastases to liver (114), Metastases to lung (73), Prostate cancer metastatic (56), Metastases to central nervous system (53), Metastases to spine (36), Metastasis (35), Bone giant cell tumor malignant (26), Metastases to lymph nodes (26), Cancer pain (21)*, Osteosarcoma (17)*, Malignant transformation (15), Metastatic neoplasm (15), Hormone-refractory prostate cancer (12)*, Metastases to meninges (11)*, Sarcoma (8)
Musculoskeletal and connective tissue disorders	Osteonecrosis of jaw (3082), Bone pain (231), Pain in jaw (167), Osteonecrosis (102), Pathological fracture (99), Exposed bone in jaw (81)*, Bone disorder (49), Osteitis (43), Jaw disorder (37), Osteopetrosis (18)*, Bone lesion (18)*, Bone lesion excision (13)*, Jaw fistula (10)*, Osteolysis (10)*, Osteonecrosis of external auditory canal (8), Bone sequestrum (8)*, Hungry bone syndrome (6)*
Gastrointestinal disorders	Tooth disorder (177), Toothache (104), Tooth loss (47), Dental caries (39), Loose tooth (38), Gingival pain (26), Periodontal disease (22), Oral cavity fistula (13)*, Gingival disorder (12), Gingival recession (9)*, Tooth resorption (6)*, Pulpless tooth (6)*
Injury, poisoning and procedural complications	Atypical femur fracture (122), Femur fracture (100), Tooth fracture (60)*, Fracture (34), Atypical fracture (30), Spinal compression fracture (28), Stress fracture (19)*, Femoral neck fracture (17), Jaw fracture (13), Traumatic fracture (10)*, Dental restoration failure (7)*
Infections and infestations	Osteomyelitis (151), Periodontitis (46), Tooth infection (45), Tooth abscess (37), Abscess jaw (33), Gingivitis (27), Gingival abscess (13), Dental fistula (10)*, Abscess oral (9), Abscess neck (7)
Investigations	Blood calcium decreased (112), Prostatic specific antigen increased (36)*, Blood phosphorus decreased (29), Blood parathyroid hormone increased (28)*, Blood calcium abnormal (27), Investigation (23)*, Tumor marker increased (19)*, Blood parathyroid hormone decreased (10)*
Metabolism and nutrition disorders	Hypocalcemia (799), Hypercalcemia (113), Hypophosphatemia (90), Tetany (30), Hypomagnesaemia (27)
General disorders and administration site conditions	Disease progression (160)*, Impaired healing (78)*, Therapy partial responder (35)*, Terminal state (23)*
Nervous system disorders	Spinal cord compression (35)*
Congenital, familial and genetic disorders	Gene mutation (12)*
Endocrine disorders	Hypercalcemia of malignancy (8)

Note: * denotes suspected ADEs of denosumab that have not been previously reported in the literature.

## 4. Discussion

### 4.1. Basic information on ADE reports

A total of 10,963 ADE reports were included in this study, with a slightly higher proportion of female than male patients. Some studies suggest that this may be related to differences in breast cancer occurrence between men and women. Breast cancer is the most frequently diagnosed malignancy in women [36]; accordingly, more ADE reports involved female patients (47.92%) than male patients (40.31%). However, 11.77% of the records lacked sex information, which could introduce bias if the missing group had a distinct ADE profile. To date, no studies have specifically examined sex-related differences in denosumab-associated adverse events during antitumor therapy, highlighting the need for future investigations with comprehensive demographic data. Among the countries that reported, the United States was the primary contributor, followed by Japan and Germany. China’s share was relatively low at 1.81%, which may be attributed to the relatively late market launch and clinical use of denosumab in China. This could also be related to the fact that some patients with cancer in China did not receive standardized guideline-recommended treatment post-surgery.

### 4.2. ADE signaling and SOC analysis of denosumab

Among the 320 signals identified by ROR, 294 were also flagged by BCPNN. This 91.9% overlap between ROR and BCPNN findings underscores the robustness of the detected signals and strengthens their reliability for downstream pharmacovigilance decisions, such as risk assessment and regulatory action. A total of 294 signals were identified as effective positive signals for antitumor treatment with denosumab, involving 27 SOCs. Among these, musculoskeletal and connective tissue disorders were reported most frequently, involving the largest number of signals. ADEs in this SOC are the most common in clinical practice, such as osteonecrosis of jaw, bone pain, pain in jaw, osteonecrosis, and pathological fracture, which are common musculoskeletal and connective tissue disorders in clinical practice. Some studies have reported cases of osteonecrosis of the jaw in patients receiving denosumab treatment, with symptoms including jaw pain, osteomyelitis, osteitis, bone erosion, tooth or periodontal infection, toothache, gingival ulceration, and gingival erosion [37,38]. Persistent oral/maxillofacial pain or delayed wound healing (> 8 weeks) following dental surgery may serve as early warning signs of osteonecrosis of the jaw. In clinical trials conducted on patients with cancer, the risk of osteonecrosis of the jaw increased with prolonged drug exposure. A total of 79% of patients with osteonecrosis of the jaw had a history of tooth extraction, poor oral hygiene, or use of dental instruments as precipitating factors. Among patients with multiple myeloma who developed osteonecrosis of the jaw while receiving denosumab treatment, 58% had a predisposing factor of having undergone invasive dental surgery [39]. Other risk factors for jawbone necrosis include immunosuppressive therapy, anti-angiogenic therapy, systemic glucocorticoid use, diabetes, and gum infections. Therefore, good oral hygiene must be maintained when using denosumab for treatment. For patients who cannot avoid dental surgery, international clinical guidelines recommend discontinuing the drug at least 4 weeks prior to surgery to promote alveolar bone healing.

### 4.3. Analysis of the ADE signal of denosumab

This study simultaneously applied the ROR and BCPNN methods for detection signal analysis, sorted by the frequency of ADE. Among them, osteonecrosis of the jaw occurred most frequently and was one of the most serious denosumab ADEs, followed by death and hypocalcemia. It impairs bone remodeling by inhibiting osteoclast activity and inducing cell apoptosis. In addition, the interruption of angiogenesis mediated by vascular endothelial growth factor (VEGF) may also predispose patients to osteonecrosis of the jaw, which is particularly evident when used to treat osteoporosis in elderly women [40].Su et al. [22]found that denosumab had 528 off-label uses, with breast cancer and prostate cancer being the most common off-label uses for this drug. Other off-label indications include arthritis, vitamin D deficiency, spinal compression fractures, gastroesophageal reflux disease, and plasma cell myeloma [21]. In clinical practice, new drug indications can be discovered by mining databases, promoting drug research and development, and improving clinical practice.

The study also found that, when ranked by signal strength, the ADE with the strongest signal strength was bone giant cell tumor, involving SOCs of neoplasms benign, malignant and unspecified (incl cysts and polyps), indicating a strong association between denosumab and bone giant cell tumor. The drug label states that the occurrence of malignant tumors or transformation is a rare risk in the treatment of bone giant cell tumor; however, no increase in the risk of malignant transformation of the primary disease has been observed in clinical practice in patients with bone giant cell tumor treated with denosumab.

This study also identified new suspected ADEs, including exostosis of the jaw, tooth fracture, osteosarcoma, bone lesions, and exposed bone in the jaw. This may be related to the fact that denosumab induces osteoblastic differentiation and subsequent bone formation [3], which requires prospective verification. When using denosumab to treat cancer, clinical pharmacists should not only pay attention to the ADEs listed on the drug label but also be aware of any ADEs that have not been promptly recorded or updated in the literature. This is to prevent medication safety risks caused by delays in updating the label, provide reasonable medication recommendations, and ensure patient safety.

### 4.4. Severe ADEs reports with denosumab

In this ADE signal detection for denosumab, there were 8, 444 reports of serious ADE, accounting for 77.02% (8, 444/10, 963) of the total. In addition to other serious medical events, the most common serious ADE reports resulted in hospitalization, accounting for 24.42% (2, 677/ 10, 963) of cases, with the highest number of reports for osteonecrosis of the jaw and hypocalcemia. The second most common type of report was death, which accounted for 12.09% of cases (1, 325/ 10, 963). Osteonecrosis of the jaw is a serious ADE associated with denosumab treatment and requires preventive dental management [41,42]. However, the fatal risk of osteonecrosis of the jaw has not been clearly established in large clinical studies. The report also noted that hypocalcemia is one of the most common ADEs of denosumab in clinical practice, and there have been reports of deaths, especially among elderly female dialysis-dependent patients. Studies have shown that The prevalence of end-stage CKD-related mineral and bone disorders is high among patients undergoing long-term dialysis [41,43]. Denosumab use is associated with decreased bone mass and an increased risk of hypocalcemia [44].

### 4.5. Limitation

Nevertheless, this study has some limitations that must be acknowledged. First, the reliance on self-reported data within the FAERS database introduces the possibility of reporting bias and incomplete information, which may affect the robustness of our findings [3,45,46]. For instance, some reports lacked the exact onset date of adverse events, precluding the assessment of their chronology and any potential drug–event relationship. Second, our analysis was inherently descriptive and could not establish a causal relationship between denosumab exposure and reported adverse events. Third, patient-level covariates such as performance status, concomitant medications, comorbidities, and prior cancer history were unavailable, precluding adjustment for potential confounders and limiting the interpretability of observed associations. Additionally, the reporting of additional malignancies could not be definitively classified as second primary cancers, disease progression, or treatment-induced neoplasms without access to longitudinal clinical and histopathological data. Future studies should address these limitations by incorporating additional databases and combining them with real-world evidence to validate and substantiate our findings. Additional studies are essential to explore the mechanisms underlying the identified ADEs and develop strategies to mitigate these risks while maximizing the therapeutic benefits of denosumab therapy. This knowledge is crucial for optimizing treatment strategies and ensuring that patients receive safe and effective care.

## 5. Conclusion

Using the U.S. FAERS database and complementary disproportionality methods (ROR and BCPNN), we delineated the frequency and profile of ADEs linked to 120 mg denosumab. These findings provide clinicians with a concise risk map for proactive prevention and prompt management during anticancer therapy. For new suspected ADE reports, clinicians should pay closer attention to these potential ADEs, strengthen the monitoring and assessment of patients, and reduce the risk of ADEs.

## Supporting information

S1 Table Example of deduplicated reports. (DOCX)

S2 Table Time to Onset of Adverse Drug Event After Administration (days). (XLSX)

S3 Table MedDRA-signal-caculations-by levels. (XLSX)

S4 Table Number of PT signals under SOC. (XLSX)
