# Supplementary material for: Mining the FAERS database reveals new safety signals for 120 mg denosumab in oncology practice
Source: PLoS One. 2026 Feb 2;21(2):e0342188. doi: 10.1371/journal.pone.0342188 (PMC12863689; doi:10.1371/journal.pone.0342188)
Supplement: S1 Table — (DOCX) [file pone.0342188.s001.docx]

Sup Table 1 Example of deduplicated reports.

| PRIMARYID | CASEID | FDA_DT | Action |
| --- | --- | --- | --- |
| 4271953 | 4070800 | 20040113 | Delete |
| 4271960 | 4070800 | 20040113 | Delete |
| 4283861 | 4070800 | 20040130 | Delete |
| 4314767 | 4070800 | 20040308 | Keep |
